# Prevention of Suicidal Relapses in Adolescents With a Smartphone Application: Bayesian Network Analysis of a Preclinical Trial Using In Silico Patient Simulations

**DOI:** 10.2196/24560

**Published:** 2021-09-30

**Authors:** Stephane Mouchabac, Philippe Leray, Vladimir Adrien, Fanny Gollier-Briant, Olivier Bonnot

**Affiliations:** 1 Department of Psychiatry Sorbonne Université Hôpital Saint Antoine- APHP Paris France; 2 Infrastructure of Clinical Research In Neurosciences- Psychiatry Brain and Spine Institute (ICM), Inserm UMRS 1127, Centre national de la recherche scientifique Sorbonne Université Paris France; 3 Laboratoire des Sciences du Numérique de Nantes Centre national de la recherche scientifique University of Nantes Nantes France; 4 Department of Child and Adolescent Psychiatry Centre hospitalier universitaire de Nantes Nantes France; 5 Pays de la Loire Psychology Laboratory EA4638 Nantes France

**Keywords:** suicide, bayesian network, smartphone application, digital psychiatry, artificial intelligence

## Abstract

**Background:**

Recently, artificial intelligence technologies and machine learning methods have offered attractive prospects to design and manage crisis response processes, especially in suicide crisis management. In other domains, most algorithms are based on big data to help diagnose and suggest rational treatment options in medicine. But data in psychiatry are related to behavior and clinical evaluation. They are more heterogeneous, less objective, and incomplete compared to other fields of medicine. Consequently, the use of psychiatric clinical data may lead to less accurate and sometimes impossible-to-build algorithms and provide inefficient digital tools. In this case, the Bayesian network (BN) might be helpful and accurate when constructed from expert knowledge. Medical Companion is a government-funded smartphone application based on repeated questions posed to the subject and algorithm-matched advice to prevent relapse of suicide attempts within several months.

**Objective:**

Our paper aims to present our development of a BN algorithm as a medical device in accordance with the American Psychiatric Association digital healthcare guidelines and to provide results from a preclinical phase.

**Methods:**

The experts are psychiatrists working in university hospitals who are experienced and trained in managing suicidal crises. As recommended when building a BN, we divided the process into 2 tasks. Task 1 is structure determination, representing the qualitative part of the BN. The factors were chosen for their known and demonstrated link with suicidal risk in the literature (clinical, behavioral, and psychometrics) and therapeutic accuracy (advice). Task 2 is parameter elicitation, with the conditional probabilities corresponding to the quantitative part. The 4-step simulation (use case) process allowed us to ensure that the advice was adapted to the clinical states of patients and the context.

**Results:**

For task 1, in this formative part, we defined clinical questions related to the mental state of the patients, and we proposed specific factors related to the questions. Subsequently, we suggested specific advice related to the patient’s state. We obtained a structure for the BN with a graphical representation of causal relations between variables. For task 2, several runs of simulations confirmed the a priori model of experts regarding mental state, refining the precision of our model. Moreover, we noticed that the advice had the same distribution as the previous state and was clinically relevant. After 2 rounds of simulation, the experts found the exact match.

**Conclusions:**

BN is an efficient methodology to build an algorithm for a digital assistant dedicated to suicidal crisis management. Digital psychiatry is an emerging field, but it needs validation and testing before being used with patients. Similar to psychotropics, any medical device requires a phase II (preclinical) trial. With this method, we propose another step to respond to the American Psychiatric Association guidelines.

**Trial Registration:**

ClinicalTrials.gov NCT03975881; https://clinicaltrials.gov/ct2/show/NCT03975881

## Introduction

The recent adoption of smartphone health applications that collect data (weight, exercises, etc) and allow the consumer to see graphs and diagrams illustrates the sociological and psychological power of digital self-care and self-management. This phenomenon is of particular importance for adolescents and young adults. However, asking patients to fill in scales or questionnaires on their smartphone instead of on a computer or a paper is not new. This is called ecological momentary assessment (EMA), a naturalistic method to access clinical data [1]. However, to be useful for users, there is a need for advice, therapeutic care, or at least personalized feedback related to the answers provided, which could be the field of ecological momentary intervention (EMI).

With the development of machine learning, it is obvious that EMI will have significant implications in the future. There are only a few experiences in psychiatry with EMI, but recent reviews suggest that results are very promising in mood disorders, anxiety, and schizophrenia [2-5]. However, those apps have different approaches. Some of them focus on providing in-app therapy, such as iBobbly [6], SuperBetter [7], Get Happy [8], or ACT Smart [9]. Few of them provide a more personalized therapy according to EMA answers from a medical team (connected device) such as MONARCA (monitoring treatment and prediction of bipolar disorder episodes) [10] or more automated like PRISM (the person real-time intervention for stabilizing mood) [11]. Except for Mindstrong [12], which uses digital phenotype (ie, no psychological data but only digital use skills such as typing or screen scrolling), none of them are using in-app artificial intelligence (AI) based on EMA data. Interestingly, and somehow counterintuitively, the efficacy of these apps for depression and anxiety is better when they are not part of a program with human interactions [2,13]. This could be an argument for more intuitive and personalized apps capable of being independent of direct care. We also know that the fill rate is higher when patients have more severe symptoms [14].

Suicide attempts (SAs) are a major health care issue and could be an interesting focus for add-on smartphone app care. Suicide is a highly challenging multifactor process encompassing genetics, psychological, social, cultural, and life-experienced trauma influences [15]. More than 25 million people worldwide attempt suicide each year, and around 800,000 people die from suicide [16]. Repeated epidemiological studies show that SA history is one of the major risks of suicide. Within a year of the SA, the repetition rate in adolescent and young adult populations ranges from 15% to 28% [17]. Rates of repetition are higher within 6 months of the previous SA [18]. To date, no suicide prevention program is better than others, and all of them require the active participation of health care professionals (eg, training, calls, interviews, etc) [19]. However, studies have shown that personalized brief contact interventions reduce recurrence after an SA [20]. A specific and personalized app could optimize these strategies.

Therefore, to prevent a patient’s SA and suicide, we have created an EMA plus EMI new approach based on a mobile health care application. Our application, which is not connected to the medical team via the internet, will collect data from the patient twice a day regarding anxiety, mood, and sleep disorders, with decreasing frequency during 1 year. The data entries by the patient will be made via analogic visual scales and drop-down lists. Then, algorithm-based feedback enhanced with AI will proactively inform the patient with comments and advice based on the World Health Organization’s recommendations or associated self-coping or mindfulness practices.

The aim is to position our smartphone application like a health care partner, which is why we named it “Medical Companion.” However, to be relevant, our app needs to encompass AI.

Many specialists are convinced that AI and machine learning will be major breakthroughs in medicine and psychiatry [21-23] and could disrupt the practices [24]. Machine learning is a computational strategy that automatically determines (learns) methods and parameters to find the best solution for a problem rather than provide a previously set solution programmed a priori by a human. The strength of machine learning is its ability to explore multiple patterns in complex data. The algorithms used in machine learning can categorize, cluster, and predict by using supervised and unsupervised techniques. The most used algorithm is the support vector machine, a multivariate supervised learning technique that classifies subjects into groups within a margin-based statistical framework.

Promising achievements have been reached by this kind of bottom-up analysis with machine learning. For example, in medical imaging, researchers used more than 1000 anonymous patient X-rays to train a naïve Bayesian network (BN) to detect tuberculosis [25]. The algorithm had close to a 100% accuracy rate. There are several recent reviews and perspectives in this field [22,26-28].

The same approach has been used in psychiatry, with a research focus on diagnosis, treatment effect prediction, and outcome prediction. Authors used physiological markers [29], neuroimaging data [30] or clinical specific scales [31] with up to 75% accuracy [30,32].

However, for optimal accuracy, machine learning needs a substantial amount of data. Contrary to biological or imaging data, clinical psychiatry produces heterogenic data often associated with poor availability and management [21,33]. Moreover, this prediction bias increases with large samples when calculated with probabilistic statistics [21,33,34]. Therefore, some authors suggest the use of more expert-based algorithms using BN [35,36]. Our goal is to create an algorithm that selects different types of pre-established advice based on each individual’s limited data.

Our algorithm, developed with this expert BN technique, will achieve a high level of personalized advice and comments based on individual answers to our in-app questions.

This paper describes the method to build the algorithm. It is part of a large trial, oriented on clinical efficiency and designed per recent American Psychiatric Association (APA) guidelines for digital devices [37]. We are currently working on the smartphone application by itself, and we will run a clinical trial to evaluate its feasibility, efficacy, and use (clinical trial NCT03975881) in real life.

## Methods

### Theoretical Background of BN

Pearl [38] defines a BN as a probabilistic graphical model that allows the representation of a set of variables and their probabilistic relationships using a directed acyclic graph (DAG). By connecting the cause to the effect with an arrow, we obtain the most intuitive graphical representation of the influence of one event, one act, or one variable on another. It models the causal relationships well. Interestingly, BNs are knowledge representation models that can be built from expert experiences. Furthermore, a BN can learn from data, updating knowledge of the status of a subset of variables when other variables (the evidence variables) are observed and perform inferences by an incremental process.

A BN is an annotated DAG that encodes a joint distribution for a random set of variables X. Formally, a BN for *X* is a pair: *B* = *[G,*Θ*]*. For a summary of notations used in the text, see Textbox 1.

The first component *G* *= (X, U)* is determined by a set of variables *X = {X_1_, X_2_...X_n_}* whose elements are called vertices or nodes and a set *U = {u_1_, u_2_,..., u_m_}* of the Cartesian product *X × X* whose elements are called arcs or edges, which represent the direct dependencies between the variables. For an arc *u = (X_i_, X_j_)*, *x_i_* is the initial end (origin), *X_j_* is the final end (destination). An arc *u* is directed, starting from *X_i_* and arriving at *X_j_*. Above all, the graph *G* encodes independence assumptions to satisfy the local Markov property—each variable *X_i_* is independent of its nondescendants given its parents in *G*.

The second component of the pair, namely Θ, represents the set of parameters that quantify the network. It contains a set of conditional probability distributions (CPD) *Θ_i_ = P_B_ (X_i_|InΠ_i_)* for each variable *X_i_*, where *Π_i_* denotes the set of parents of *X_i_* in *G*.

**Notations used in the text and their meaning.**

- *B:* Bayesian network
- *G:* Graph
- *Θ:* Set of parameters that quantifies the network
- *X:* Node variable in the model
- *Π_i_*: Set of parents of X_i_
- *U:* Set of edges
- *u:* An arc

The BN defines a unique joint probability distribution over X given by:




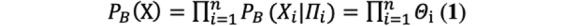




In BNs, conditional probability tables (CPTs) associated with each node should be defined to measure the relationships between variables. However, it has been pointed out that it is usually difficult to quantify the CPTs due to the complexity of the BN, which is defined by its dimension (ie, the number of independent parameters used to describe its CPD).




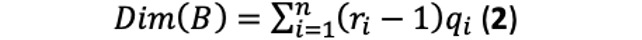




where *r_i_* is the cardinality of *X_i_* and 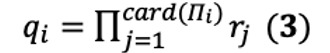 is the number of configurations of the parents of *X_i_*. If X_i_ has no parents, *q_i_*=1.

So, if a binary variable has *n* binary parents, the corresponding contribution q_i_ of its CPD to the model complexity is exponential and equals 2^n^, since each *r_j_*=2. Specifically, the elicitation of all BN probabilities is a complex and time-consuming task. To simplify a CPT, we can use a noisy-or model (NOM) where the number of probability values to estimate is now proportional to the number n of parents. Thus, it allows for simplifying the CPD [38].

### Building a BN From Expertise

The knowledge bases are built to formalize our knowledge in specific domains and support our reasoning on events and decisions in a structured way. A BN is a declarative (“knowing what”) knowledge-representation formalism constructed from expertise. Due to its probabilistic content, it allows for exploiting more efficiently the structure of the knowledge bases.

Constructing a BN from expertise is based on several steps. For this purpose, Kjaerulff and Madsen [39] divide the process into 2 tasks: (1) structure determination, representing the qualitative part of the BN, and (2) parameter elicitation, with the conditional probabilities, corresponding to the quantitative part.

Determining the structure requires skills, inventiveness, close communication with experts, and a high level of expertise. This process needs to address 2 main tasks: (1) identification of the relevant variables and (2) identification of the links between the variables.

The parameter elicitation makes the tacit knowledge as explicit as possible (and therefore easier to convey) and formalizes the expert's reasoning in an inference engine. It aims at eliciting subjective conditional probabilities from expertise to artificially reproduce the analysis of the situation and the decision-making of the expert. Finally, expert domain knowledge can be handled as prior distribution, but it is not certain that the expert has the experience to formulate valid probability judgments naturally. Authors point out that the experts must be assisted by an experienced probabilistic facilitator who will provide feedback to the experts, for example, by using simulations [40]. Inconsistencies will be discussed and will allow the model to be adjusted. Inversely, the facilitator needs a clear understanding of the decision problem for which an elicited probability distribution is required.

Many elicitation methods exist, but the most popular is the roulette method (or the “chips and bins method” or histogram method) [41]. It is prized by the experts because it provides visual feedback in the form of histograms.

### Building Our BN

Our project (clinical trial NCT03975881) is an innovative approach to prevent the relapse of SAs and suicides in patients with a previous SA. It is the core of our healthcare smartphone application. The program is an add-on to the usual care process and is built to work autonomously. The BN will help to:

1. Estimate actual psychiatric and behavioral state based on users' answers to in-app questions.
2. Match the estimated state with appropriate and accurate preexisting advice and care provided by the smartphone app.

#### Variables Determination for Estimating Behavioral and Psychiatric State

At first, we have identified contextual variables that are mandatory data in any clinical study (ie, age, gender, type of SA, date, and localization). Only age and gender will be exploited in the algorithm to reduce the required amount of expert estimation. Then we defined clinical questions related to the mental state of the patients. All questions (Q) have been provided by experts in the form of a single choice and are related to specifics factors. The elicitation of each clinical dimension corresponds to a set of questions Q = {Q_1_…Q_m_}.

The clinical dimensions were chosen for their known and demonstrated link with the suicidal risk in the literature [42,43]. Some are clinical markers related to depression (mood, cognitive retardation, sleep or appetite disorders, and physical pain) or anxiety, as well as a measure of the presence and the severity of suicidal ideation. The intensity measures of suicidal thoughts were adapted from specific psychometric scales (ie, the Columbia-Suicide Severity Rating Scale) [44]. Others are behavioral markers that can be disturbed if the patient's clinical condition changes (eg, the use of video games) or may promote suicidal ideation (eg, substance use disorder).

We had 9 clinical dimensions, 23 questions, and 10 pieces of advice. All questions were linked according to clinical knowledge and translated into the BN model. Categories were anxiety, sleep, appetite, physical complaints (eg, headache, stomach pain, etc), cognitive impairment (concentration and memory), mood, suicidal thought and behavior, and addictive behavior (eg, alcohol, tobacco, screens, and games, etc).

To simplify and fit a four-level Likert scale, an expert group created the questions to approach the mental state category. For example, “How anxious are you?”, “How much were you able to cope with your anxiety?” and “How long were you anxious during the last day?” For the last question, instead of “absent,” “low,” “medium,” or “high,” we opted for “none,” “less than 1 hour,” “from 1 to 3 hours,” and “almost all day.” All questions were a priori scored by an expert according to the BN structure.

All patient answers were made of visual analog scales (VASs). The formulated questions were slightly modified every day (and twice a day during the first weeks) to avoid a perception of a process that seems too “automatic.”

More precisely, the discussions between the experts led us to identify 3 variables for each factor, described as follows:

1. Immediate value of factor (IF) corresponds to the measure in percentage extracted from the questions for the VAS at instant *t*.
2. Cumulated value of factor (CF) is the mean of time-repeated immediate value. If we consider that the weight given to each datum is equivalent, the more the data increases, the lower the weight of each datum, which will decrease the responsiveness of the system (ie, the anxiety experienced several weeks ago might not have the same impact for calculating an estimated state than a few hours ago). To address this issue, we have introduced a forgetting factor α to reduce the weight of older data as follows:
  CF_new_ = (1-α) × IF + α × CFold
  If α=0, no aggregation, CF=IF; if α=1, no update, the CF remains unchanged.
3. Contextual severity (CS) corresponds to a discrete variable with a domain (high, medium, low, absent) that describes the seriousness of one given dimension with respect to one given context (which is the CF describing the user characteristic). For instance, for some dimensions, a high IF is “severe” when the CF for this dimension is also high, but it is less serious when the CF is low (ie, if a patient anxiety state is “none,” a single high anxiety score stays relatively low compared to a patient who is repeatedly anxious, independent of their level of immediate anxiety).

#### Structure Determination of the BN

**Figure 1:**
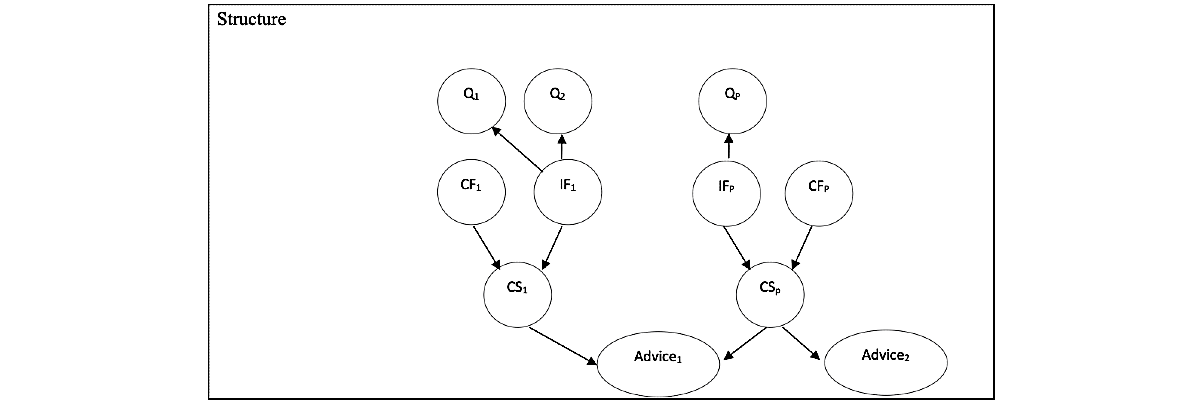
Bayesian Network Structure. CF: cumulated value of factor; CS: contextual severity; IF: immediate value of factor; Q: question.

The BN structure (Figure 1) models the expert way such as:

1. The state regarding one dimension can vary according to the patient context, but as contextual variables, only age and gender are included in the model.
2. The patient will not answer a question regarding his state similarly for the corresponding dimension (eg, answers concerning anxiety will be different if he is usually very anxious vs not anxious).
3. The severity of one specific dimension depends on a “comparison” between its IF and CF, confronting temporal aspects of states.
4. Each advice depends on the severity of potentially several dimensions because it could be triggered by one or many dimensions.

The CS of a specific dimension depends on both IF and CF. The combination of different CS from a selected set of dimensions gives the most efficient advice related to those dimensions. Nodes are represented as circles and edges as arrows. Responses to each question (Q) associated to each dimension depend on the IF of the dimension, and they become independent of each other if we know the value of IF.

#### Parameters Elicitation

According to the 2-task process described by Kjaerulff and Madsen [45], we identified from the previous structure the following parameters (ie, CPD) to validate the work of our experts. Due to the high number of dimensions used in this work and the large volume of data, we chose to illustrate this part only with a selected factor, anxiety, because it is a dimension whose severity can change very acutely. Therefore, we can illustrate the model’s reactivity to an acute change of clinical state. The same has been applied to each dimension.

For each dimension *I*, we defined an a priori probability as follows: *P(IF_i_=high)*, *P(IF_i_=medium, P(IF_i_=low)*, and *P(IF_i_=absent)* so that a “random” patient will be associated with this dimension to the corresponding level (high, medium, low, and absent) regarding his context (age and gender). *P(CF_i_)* will be initialized to *P(IF_i_).*

For each question related to its dimension, *P(Q = j/IF_i_ = k )* is the probability that the answer of this question Q will be the *j*th *one*, given that the patient IF is in its *k*th level.

The main issue is to estimate how a specific individual with specific clinical characteristics may answer a question about his specific state with sufficient accuracy. However, there are no epidemiological or clinical works available with such a level of detail regarding the studied population. Thus, it is impossible to refer to scientific data for the percentage of patients with a medium level of anxiety answering low anxiety to a question asked. Through their knowledge and experience, experts may be able to provide accurate answers. To estimate the value in the domains (absent, low, medium, and high), we use probability calculations as follows:

- *P(CS_i_/IF_i_,CF_i_)*: Defined the severity given the dimensions’ IF and CF.
- *P(advice/CS_1_...CS_p_)*: The scoring depends on all the dimensions’ severity, specifically, CS, and as previously described in the theoretical background section of BN, a NOM distribution is used to simplify this dependency.

Experts also offered advice addressing many efficient actions for suicidal crisis management such as lifestyle, sleep, relaxation or mindfulness exercises, management of social rhythms, physical activity, and emergency solutions (ie, calling person in the patient’s pre-established circle of confidence, exercising with abdominal breathing, practicing mindfulness or relaxation, and calling emergency services) [44].

Each piece of advice might be useful for every factor but at different levels of accuracy. Therefore, the advice must have a good sensitivity (ie, if it is essential, the system must not omit it). Similarly, if it is not essential given the patient's state, it should not be triggered in excess, so it must have good specificity. For this purpose, experts have estimated the a priori probability *P(advice/ CS_1_...CS_p_)* of advice efficiency due to the intensity of each factor.

#### Algorithm Accuracy Testing by Simulation

##### Step 1

Building the algorithm is a multistep process. First, we perform a simulation of the first data set for mental state provided by the expert (Table 1) using the BN simulation model. Our model generates three distributions of factors: (a) a priori, (b) when all answers associated with the factor are the highest and have very severe scoring P(++), and (c) when all answers associated with the factor are the lowest and have less severe scoring P(--). Simulations are made for each category of mental state.

**Table 1:** Expert-based Bayesian network construct regarding anxiety.

What is your level of anxiety?	Distribution for 100 subjects with no (absent) anxiety	Distribution for 100 subjects with low level of anxiety	Distribution for 100 subjects with medium level of anxiety	Distribution for 100 subjects with high level of anxiety
No anxiety	100	5	0	0
Low anxiety	0	90	15	5
Medium anxiety	0	5	80	15
High anxiety	0	0	5	80
Total	100	100	100	100

##### Step 2

Return to the expert with simulation results. If simulations are accurate regarding medical expert knowledge, step 3 is possible. If not, experts provide a new data set, and we rerun step 1.

##### Step 3

Next, we perform a simulation of the first data set for the advice provided by experts using the BN simulation model (Table 2). Our model generates incremental updates (1 to 15 successive scorings by patients) of (a) IF in a specific pattern of the first 9 successive scorings at the lowest score, (b) CS values (based on CF) in a specific pattern of the first 9 successive scorings at lowest score then, IF in a progressive increasing severity pattern from scoring 10 to 15, (c) CS values (based on CF) in a progressive increasing severity pattern from scoring 10 to 15 then, IF for an immediate highest severity scoring starting at answer 10 lasting until answer 15, (d) CS values (based on cumulated severity) for an immediate highest severity scoring starting at answer 10 lasting until answer 15, and (d) for each of the 1 to 15 scorings in both patterns (ie, immediate or progressive), our algorithm will generate the advice and recommendations.

**Table 2:** Conditional probability of contextual severity as a function of the immediate value and cumulated value of a given factor.

CF^a^ and IF^b^			Contextual severity function of CF and IF						
			High		Medium		Low		Absent
**High**									
	Medium	0.98		0.02		0		0	
	High	0.99		0.01		0		0	
	Low	0.9		0.1		0		0	
	Absent	0.85		0.15		0		0	
**Medium**									
	High	0.19		0.8		0.01		0	
	Medium	0		0.99		0.01		0	
	Low	0		0.71		0.28		0.01	
	Absent	0		0.66		0.3		0.04	
**Low**									
	High	0.11		0.23		0.65		0.01	
	Medium	0		0.19		0.8		0.01	
	Low	0		0.01		0.98		0.01	
	Absent	0		0.01		0.7		0.29	
**Absent**									
	High	0.05		0.15		0.3		0.5	
	Medium	0		0.12		0.23		0.65	
	Low	0		0.01		0.19		0.8	
	Absent	0		0		0.01		0.99	

^a^CF: cumulated value of factor.

^b^IF: immediate value of factor.

##### Step 4

Different values and their distribution along the train of different pre-established scoring are presented to experts for review. They are asked to examine the accuracy and consistency of the advice proposed compared to their clinical expertise and knowledge. Then, if necessary, changes are made in the model, and the simulation is rerun beginning with step 3. Reviews and reruns are made as many times as necessary to achieve our goal. Expert opinion is a qualitative variable (yes or no).

## Results

Simulations were done according to our 4-step methodology. For each run, experts provided estimations regarding the distribution of answers in specific populations (Table 1). Experts are asked to estimate for 100 subjects with a specific state regarding anxiety (absence, low, medium, or high) how they should answer, and what would be the distribution of answers (no, low, medium, or high) because not all highly anxious patients will submit a high anxiety rating. This corresponds to *P(answer(Q)/IF_i_)*.

At each run, we calculated the conditional probability of CS as a function of the IF and CF of a given factor (Table 2). For example, if the IF and CF are high, the probability that CS will be high is 0.99 (expressing few changes). But if IF is low, with a high CF, the probability that the CF remains high is 0.9 and zero for low and absent anxiety, respectively.

**Figure 2:**
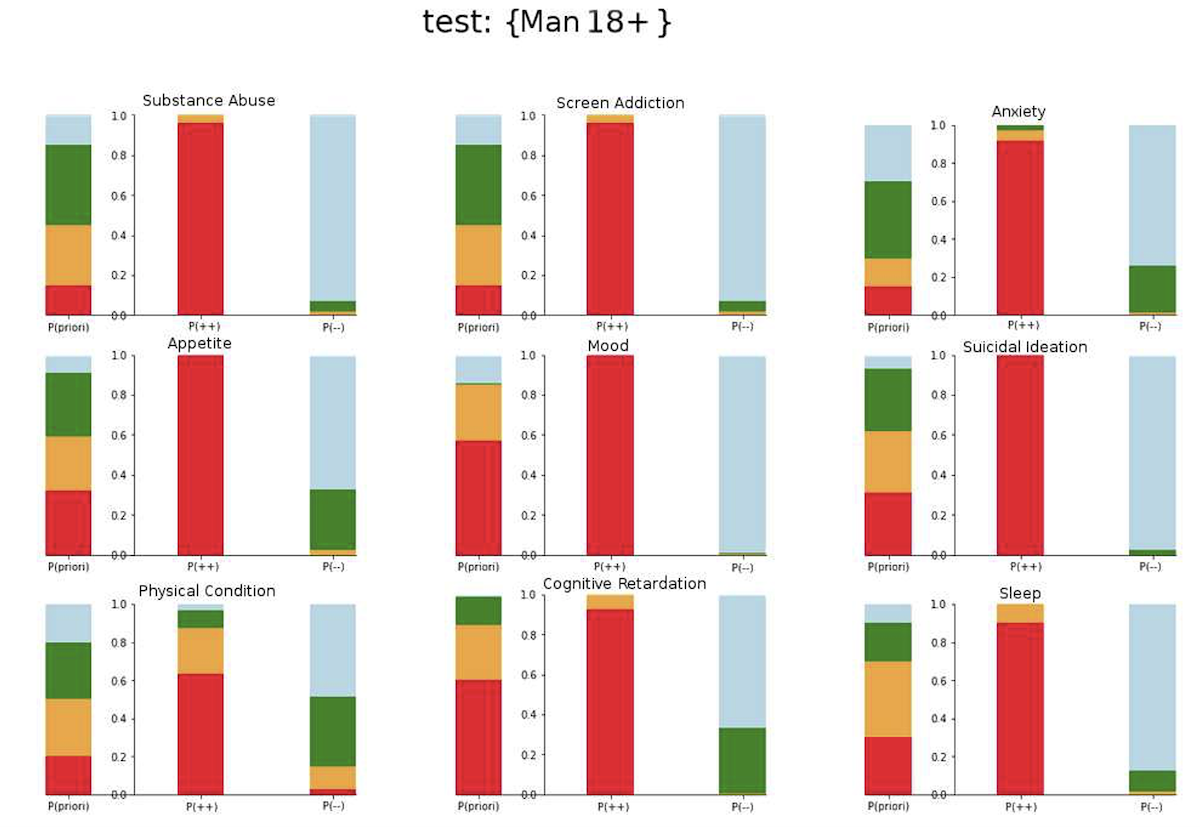
Distribution of probabilities for each dimension, for a priori status (P), high scoring P(++), and absent scoring status P(--).

The distribution of dimensions can be seen in Figure 2. After two simulation runs, the experts found that the advice proposals were accurate. The a priori status is a distribution of all 4 severity levels (absent, low, medium, and high) as proposed by the experts. In the two extreme conditions (lowest and highest severity), the experts are far less heterogeneous and become more accurate regarding clinical knowledge. Priori denotes the expert estimated distribution of answers by severity in our population of patients with previous SAs (absent in blue, low in green, moderate in orange, and severe in red). A P(++) rating of users’ scores illustrates a severe rating for all questions related to anxiety, and we calculated the algorithm-generated score for each related category of mental state. A P(--) rating of users’ score demonstrates the lowest rating for all questions related to anxiety, and we calculated the algorithm-generated score for each related category of mental state.

In the second phase, the main goal was to achieve an accurate match between all scoring by categories and the advice. The experts did provide a priori distribution of answers according to the BN structures (Table 3). For example, the first line means “what is the a priori probability that the immediate state is high, medium, low, and absent knowing that the person is a man aged above 18 years.

**Figure 3:**
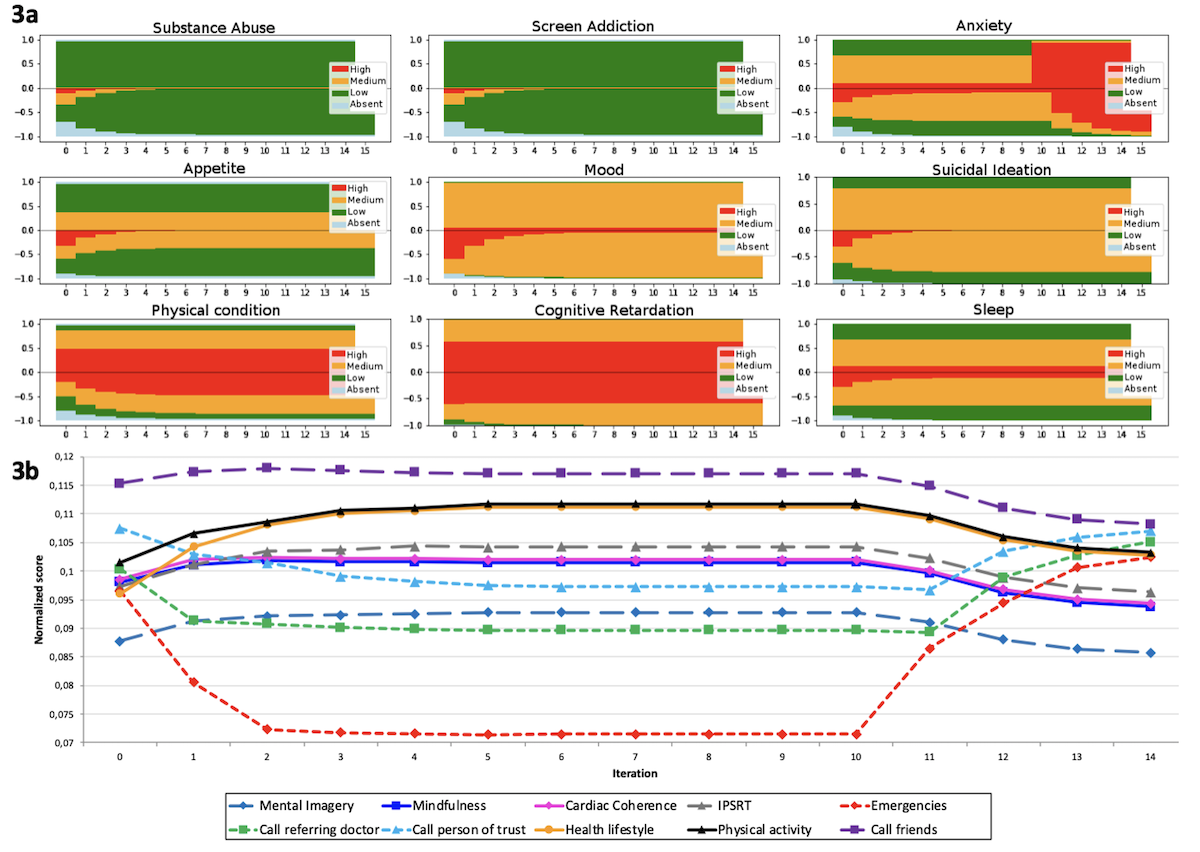
Final run simulation of the probability of advice proposal during incremental updates (immediate worsening after 9 successive lowest scores). IPSRT: interpersonal and social rhythm therapy.

**Figure 4:**
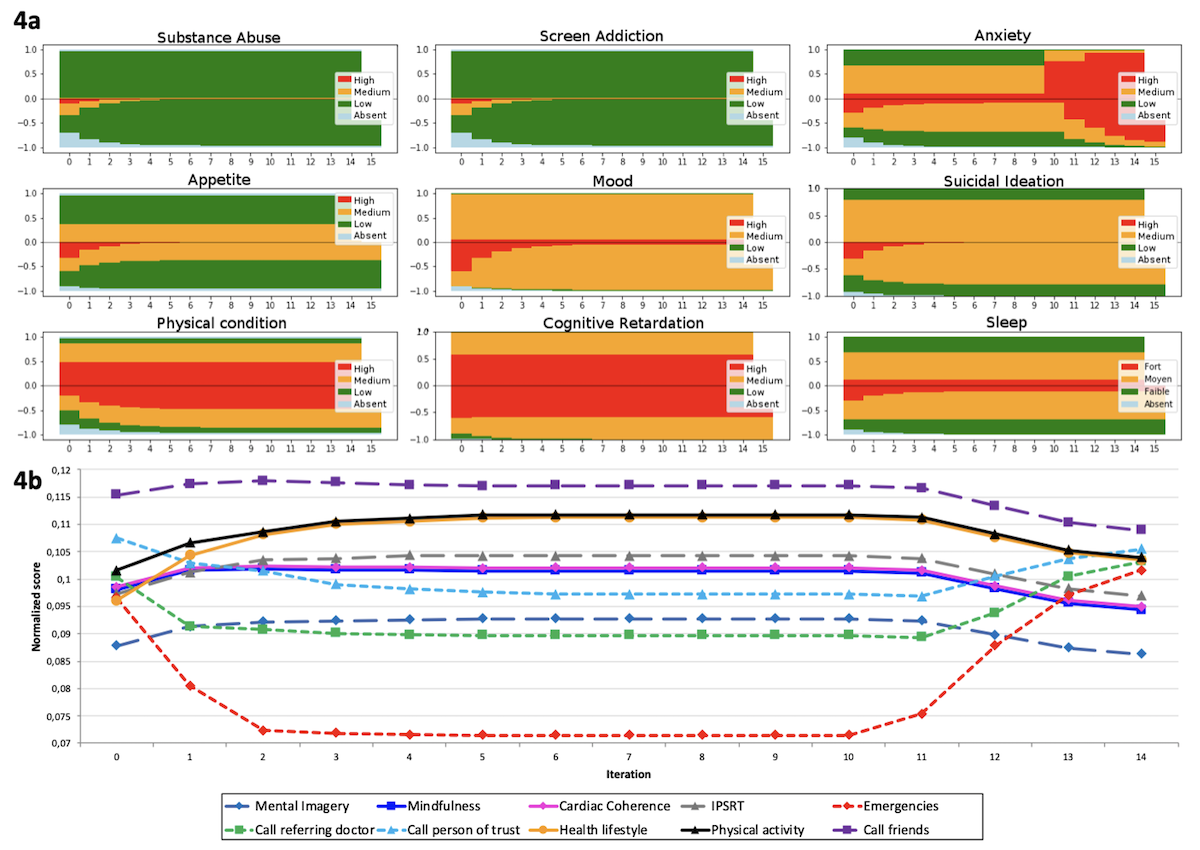
Final run simulation of the probability of advice proposal during incremental updates (immediate worsening after 9 successive lowest scores). IPSRT: interpersonal and social rhythm therapy.

The final simulations of matching answers and advice for anxiety are provided in Figure 3 and Figure 4, and an example of an expert-based BN construct is shown in Table 4. Figure 3 represents the final run simulation of the probability of advice proposal during incremental updates of 15 successive scorings by virtual patients according to an immediate worsening after 9 successive lowest scores. In Figure 3a, for each dimension, ordinate scores above zero correspond to IF and below zero CS values (based on CF). Moreover, in Figure 3b, the normalized score of the 10 pieces of advice related to anxiety are represented over the 15 steps of the simulation. Figures 4a and b show the final run simulation of the probability of advice proposal during incremental updates according to a progressive worsening after 9 successive lowest scores. Table 4 demonstrates an expert’s BN construct regarding the “call friends” advice and their accuracy in categorizing patients with various anxiety levels. For example, this specific advice might be relevant for 50 out of 100 patients with low anxiety level and 99 out of 100 with high anxiety level. This corresponds to P(advice = call friend/CS_anxiety_). This construction has been made for each piece of advice.

**Table 3:** Probability distribution for anxiety of the immediate value and cumulated value a priori based on age and gender.

			High		Medium		Low		Absent
**Men, age (years)**									
	>18	0.15		0.15		0.4		0.3	
	<18	0.2		0.2		0.35		0.25	
**Women, age (years)**									
	>18	0.28		0.28		0.22		0.22	
	<18	0.3		0.3		0.2		0.2	

In the first part of each figure (Figure 3a and Figure 4a), IF and CS for each dimension are represented. Above zero is the a priori distribution of IF; below zero reflects the progressive increase of knowledge (learning ability) represented as CS becoming progressively the mirror of IF over time.

In the second part of each figure (Figure 3b and Figure 4b), advice suggestion probabilities are initially fixed in the proportions of a general population of patients that committed SA independent of their clinical state. By increasing the knowledge of the specific clinical state of a given patient (ie, with a low and constant level of anxiety), the advice is adapting and stabilizing. When anxiety IF scoring becomes suddenly (3) or progressively (4) severe, some of them are rising (eg, emergencies and calls to referent psychiatrist or the person of trust), and others are decreasing, finally stabilizing again in another configuration.

The simulation stops when the advice has the same distribution as the a priori state and the advice is clinically relevant. After two simulation runs, experts found accurate matching.

**Table 4:** Expert-based Bayesian network construct regarding “call friends” advice.

Contextual severity: call friends, social contacts (circle of proximity)	Anxiety		
	High	Medium	Low
Yes	99	80	50
No	1	20	50

## Discussion

### Principal Findings

Our results are consistent with our hypothesis and strongly suggest that BNs are an interesting model for developing algorithms in highly expert professional fields, especially when data are unavailable.

Our model showed that depending on the clinical dimensions of a patient with a stable clinical state, it will rapidly converge (in three iterations) to a pattern of pertinent advice. In the example described in Figure 3 and Figure 4 for a patient with a constant and low level of anxiety, the pattern of advice converges to a solution that inputs priority advice without caregivers’ interventions. Indeed, going to the emergency and calling the referent psychiatrist or a person of trust are not pertinent advice for this level of severity. When the clinical state worsens, the model still demonstrates a rapid adaptation and stabilization to this event. The advice pattern reorders itself pertinently by prioritizing emergencies and caregiver intervention, and other forms of advice such as mindfulness or therapy become less pertinent. These less pertinent types of advice decrease over time when the clinical state worsens (Figure 3 and Figure 4). They are not directly dependent on the severity of anxiety, and their absolute probability stays constant. However, their relative probability (ie, the scores after normalization across every form of advice) decreases over time.

We also see that the weight of some advice, such as mindfulness and cardiac coherence, are the same. This might be explained by the fact that only the weight linked to cognitive retardation is different. Cognitive retardation does not play a role in a scenario on anxiety (Figure 3 and Figure 4), showing that its level of contextual severity stays the same over time and is independent of the level of anxiety.

As we expect, such an a priori expert model is valuable to test preclinical situations, and the results of our simulation with various patient profiles allow us to build a valid BN. The APA points out that application developers often make many claims, while the level of scientific evidence that accompanies these apps is often quite low [37,46].  In a recent study [47], the authors analyzed the claims of 73 mental health applications (the top-ranked applications from the two largest app stores) for acceptability and efficiency. There was a difference between positive statements about their effectiveness and acceptability. They found that less than 53% of these claims were associated with evidence in the scientific literature, and 33% referred to techniques for which no evidence could be found.

For this purpose, the APA proposes a hierarchical framework of 5 levels to evaluate apps [39]. The first level of the model aims to evaluate the quality of the information referenced by the application to decide if we can consider using the app. Scientific data is very important in this step. Levels 2 and 3 constitute the basic medical decision-making process centered on nonmaleficence. As in any therapeutic intervention or evaluation, apps induce risks that must be scientifically evaluated but are often overlooked; that is the goal of the second level.  Level 3 aims to evaluate the necessary scientific evidence, providing us reasons for potential use. Level 4 evaluates the usability of the apps and level 5 tests interoperability.

This framework highlights the need for the scientific development of applications that correspond to the standards of therapeutic research. For this reason, we consider that a preclinical phase is essential before continuing to the clinical phase.

During the design phase of the device, the preclinical step makes it possible to check its performance and safety and will help us evaluate its later acceptability for the patient. By comparison, when it comes to the development of a drug, this phase makes it possible to evaluate a molecule in cultured cells (in vitro) and in animals (in vivo), but in our case, we can talk about evaluation in silico that is often used in recent pharmacological studies [48].

With our Bayesian approach, we can make simulations of patients, considering these to be preclinical tests as recommended by good practices in clinical therapeutic research, and we can increase its scientific validity. Nevertheless, it still is a simple model with only 9 clinical dimensions and 10 types of advice driven by 23 questions. In developing preclinical tests in the future, models will have to be improved with more variables.

There are numerous healthcare apps in mobile app stores. At the moment, very few provide academic validation with state-of-the-art clinical trials. To date, there are only 2 FDA (Food and Drug Administration)-approved or CE (Conformitè Europëenne)-approved apps (reSET@ and Flow) in psychiatry [49]. One provides digitalized depression prevention treatment [50], and the other is for opioid addiction management [51].

Our project is one of the first attempts to build autonomous care support for suicide prevention in a specific at-risk population of patients with previous SA with a methodology in accordance with APA guidelines.

From a larger point of view, the main idea driving this project is that it is possible to build an app that mimics a doctor’s care enough to be a valuable (and efficient) add-on. The empowerment of patients is a vital topic, and economic issues are also important.

But most importantly, we are showing that algorithms for clinical and behavioral disturbance in psychiatry might be a crucial field for BN, particularly when there is not enough available data to build such algorithms. For example, there is important scientific literature for anxiety in patients with depression, suicidal ideation, or SA; however, the data are not specific enough to answer very specific questions regarding sleep disturbance according to the level of anxiety in a patient 3 months after SAs. Therefore, expert opinion is crucial.

Such an algorithm, embedded in smartphone apps, could be used by many patients and consequently produce a large amount of new specific data. This data will eventually help us improve our model by incrementally updating the parameters (ie, all the probability distributions) to recognize one given factor better or associate one advice type better to some situations.

Since we will have a lot of usage data, it is important to determine if the recommendations are followed by action and if this type of algorithm increases their relevance. This could be of particular interest for therapeutic compliance or even access to emergency care. The clinical stage of our research, associated with our application's usage data, will allow us to study this question.

### Conclusion

We are convinced that using digital devices with efficient algorithms is crucial for medical treatment in terms of reliability and safety. However, to date, very few devices meet accurate methodological requirements. Our work is a proof of concept that emphasizes the need for preclinical trials by algorithm development. Additionally, it shows that BN is an accurate and very efficient branch of AI in psychiatry and clinical psychology.

However, building the application and randomized controlled clinical trials are necessary to confirm our choices and the overall efficacy of our device.

## Abbreviations

AI: artificial intelligence
APA: American Psychiatric Association
CF: cumulated value of factor
CPD: conditional probability distribution
CPT: conditional probability table
CS: contextual severity
DAG: directed acyclic graph
EMA: ecological momentary assessment
EMI: ecological momentary intervention
IF: immediate value of factor
NOM: noisy-or model
SA: suicide attempt
VAS: visual analog scale
